# The association between loneliness, social isolation, and sleep disturbances in older adults: A follow-up study from the Swedish good aging in Skåne project

**DOI:** 10.1177/20503121231222823

**Published:** 2024-01-18

**Authors:** Henrik Ekström, Markus Svensson, Sölve Elmståhl, Lena Sandin Wranker

**Affiliations:** The Department of Clinical Sciences in Malmö, Division of Geriatric Medicine, Skåne University Hospital, Lund University, Malmö, Sweden

**Keywords:** Older adults, loneliness, social isolation, sleep disturbances, prospective study

## Abstract

**Objectives:**

The aim of this follow-up study was to investigate whether loneliness and social isolation in a sample of older adults, mean age of 67.4 years at baseline examination, were associated with sleep disturbances at re-examination at a mean age of 76.4 years.

**Methods:**

The study sample consisted of 2897 participants. Data on loneliness, social isolation, and sleep disturbances were collected through questionnaires and medical examinations. Logistic regression models were constructed to identify associations between levels of loneliness and social isolation at baseline and sleep disturbances at follow-up. Sociodemographic and health-related confounding factors were controlled for in the models.

**Results:**

Sleep disturbances were reported by 25.6% (95% CI: 24.0%–27.2%) at baseline and 23.7% (95% CI: 22.1%–25.3%) at re-examination. Odds ratios for sleep disturbances at re-examination in relation to not being lonely or socially isolated were as follows: single occasions of loneliness (OR: 1.37, 95% CI: 1.05–1.78), recurring periods/constant loneliness (OR: 1.92, 95% CI: 1.01–1.99), less severe social isolation (OR: 1.18, 95% CI: 0.78–1.79), and severe social isolation (OR: 1.88, 95% CI: 1.01–3.49).

**Discussion:**

Sleep disturbances are common among older adults and are associated with loneliness and social isolation. Healthcare professionals should be aware of the potential effects of loneliness and social isolation when investigating sleep disturbances in older adults.

## Introduction

Loneliness can be described as a subjective emotional experience of distress associated with the feeling of having an unsatisfactory quality and/or quantity of social relationships.^1^ Social isolation is often considered an objective lack of social contact.^2^ Loneliness and social isolation have been associated with a deterioration in health and increased mortality among older adults.^3^ Although related, loneliness and social isolation are often considered separate concepts, as lonely people are not necessarily socially isolated and vice versa.^4,5^

A higher prevalence of both loneliness and social isolation is reported with increasing age. In an overview of different European populations, loneliness in the 60–79 year age group was estimated as 20%–35% and as 40%–50% in the group aged 80 years and older.^6^ Social isolation in older Swedish adults has been estimated as 5% in the 65–74 year age group, 11% in the 75–84 year age group, and 15% in the 85 years and older age group.^7^ Sleep disorders among older adults are a global challenge that is expected to increase with an aging population.^8^ A study investigating the prevalence of five sleep complaints, namely trouble falling asleep, waking up, waking too early, needing to nap during the day, and not feeling rested, in older American adults 65 years and over revealed that about 55% suffered from at least one chronic complaint,^9^ although increasing age does not necessarily imply impaired sleep.^10,11^

Disturbed sleep with too little time for recovery has been suggested to disrupt normal endocrine, metabolic, and neurological processes^12^ and is linked to deteriorating health,^13^ reduced quality of life,^14^ and increased mortality.^15^

It should also be mentioned that several other risk factors for sleep disorders and disturbances have been reported, including female gender,^16,17^ poor financial status,^18^ widowhood,^19^ preclinical cognitive decline,^20^ depressed mood,^13,21^ respiratory symptoms, and cardiovascular disease,^13^ as well as cancer.^16^

The results of recent prospective studies aimed at investigating the relationships between loneliness, social isolation, and sleep quality in older adults have varied. For example, in a 2-year follow-up, it was shown that loneliness, but not social isolation, predicted sleep quality.^22^ However, another study over a 6-year period demonstrated that social isolation but not loneliness predicted poor sleep quality.^23^

Hence, as few prospective studies have examined loneliness and social isolation as predictors of sleep disturbance in older adults, the aim of the present study was to investigate whether loneliness and/or social isolation were associated with sleep disturbance in a follow-up among older adults aged 60–93 years at baseline.

## Materials and methods

### Healthcare context

In Sweden, the healthcare system is decentralized and managed by 21 regions. Healthcare is mostly financed using taxes and to a lesser extent through private insurance.

Every region is responsible for its healthcare resources and, as a result, the healthcare services available may vary between regions. In the county of Skåne, which is the southernmost region of Sweden with both larger urban areas and rural areas, there is a population of 1.4 million (about 10% of Sweden’s population), of which 30% have a foreign background. Healthcare in the county is of a high standard and includes both primary care through contact with a general practitioner and highly specialized hospital care.^24^

Participants in the present study were drawn from the ongoing longitudinal Good Aging in Skåne (GÅS) study, which is part of the “Swedish National Study on Aging and Care (SNAC).”^25,26^ The GÅS study started in 2001 and recruited participants from five municipalities in Skåne, covering both urban and rural areas. The participants were randomly selected from the population register and invited to participate in the study by letter or, if necessary, by phone. Written informed consent was obtained from all participants. The design of the GÅS and the SNAC is described in more detail elsewhere.^25,26^

### Inclusion and exclusion criteria

Inclusion criteria were as follows: over 60 years old in the first-wave examination, either 60 or 81 years old in the second-wave examination, and resident in one of the following five municipalities in the county of Skåne: Malmö, Ystad, Eslöv, Osby, or Hässleholm. There was no predefined proportion or percentage of participants. The number of individuals invited to participate in the studies was calculated based on the clinic’s capacity and the time at our disposal.

Those who died within 90 days of the baseline examination or re-examination were considered to have been in such poor health that they would have been unable to participate in this comprehensive survey including several questionnaires, extensive medical and psychological interviews, and physiotherapeutic examinations, and were therefore excluded. The time limit of 90 days was set arbitrarily but judged to be reasonable. Other exclusion criteria were moving away from the five municipalities under study, being unable to understand written or spoken Swedish and being impossible to contact by letter or telephone.

### Study sample

**Figure 1.**
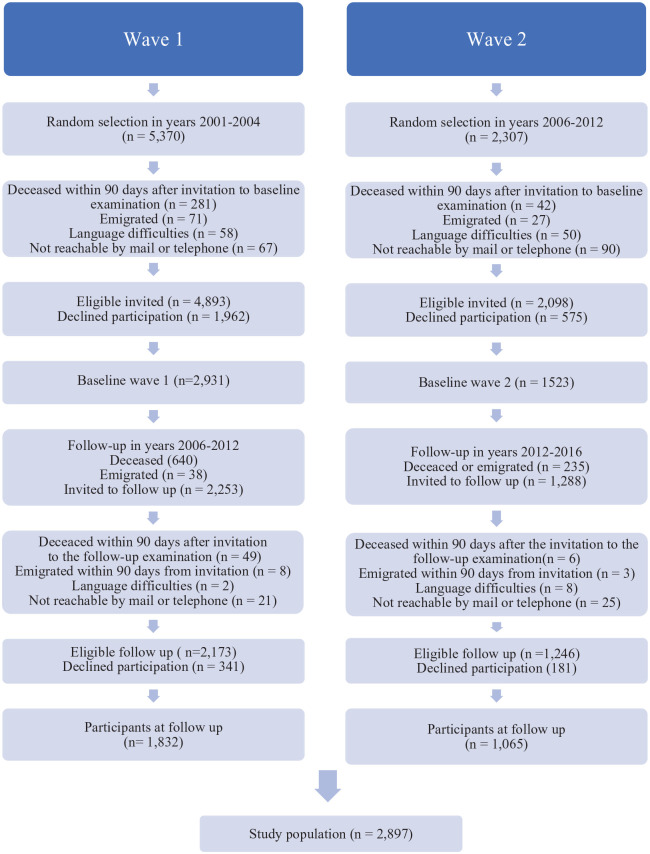
Flow chart of the inclusion in the longitudinal general population GÅS study.

In the present study, 6991 eligible individuals were invited in two waves. In the first wave, recruited from 2001 to 2004, 2931 (60.0%) out of 4893 individuals agreed to participate. In the second wave, recruited from 2006 to 2012, 1523 (72.6%) out of 2098 individuals agreed to participate. The first wave included participants in nine age cohorts (60, 66, 72, 78, 81, 84, 87, 90, and 93 years), of whom 1832 (62.5%) were re-examined between 2006 and 2012. The second wave included participants in two age cohorts (60 and 81 years), of whom 1065 (69.9%) were re-examined between 2012 and 2016. The present study sample consisted of those who took part in both the baseline examination (mean age 67.4 years) and the re-examination (mean age 73.4 years), resulting in a final sample of 2897 individuals (Figure 1).

#### Sociodemographics, loneliness, social isolation, health status, and sleep symptoms

Structured interviews, including questions about diseases and a neuropsychological test, were carried out by trained medical staff in accordance with predefined research protocols. Self-reported questionnaires were used to obtain data on sociodemographics, financial status, pain, loneliness, social isolation, sleep duration, sleep medications, and sleep symptoms. Although no psychometric analysis of the target sample was performed, well-known and well-documented instruments (in Swedish) previously used in the GÅS population study were employed. Assessments took place at the research center or, for health reasons, in the homes of the participants.

#### Sociodemographics

Sociodemographic data included age, sex, education, cohabiting status, financial status, and smoking habits.^27^ Level of education was dichotomized into elementary school (9 years of compulsory studies) and high school/college (more than 1 year of optional studies after elementary school). Marital status was dichotomized into married/cohabitant and unmarried/divorced/widowed. Financial status was assessed as good or poor, based on whether the participants answered yes or no to the question “Have you had difficulties making ends meet when it comes to running expenses during the past year?” Smoking habits were categorized as never smoked, former smoker, or current smoker.

#### Loneliness and social isolation

Loneliness was assessed by the single-item question: “When you look back at the past 3–5 years, which alternative fits you best?” The four response alternatives were as follows: (a) no feelings of loneliness, (b) I have experienced occasional feelings of loneliness, (c) I have experienced recurring periods of feelings of loneliness, and (d) I have lived with a constant feeling of loneliness.^27,28^ The two latter response alternatives were merged into one category in the analysis: recurring periods/constant loneliness.

Social isolation was assessed by a single-item question: “How often have you met your, A. Husband/wife (If not cohabiting), B. Parents, C. Children, D. Son-in-law/daughter-in-law, E. Grandchildren, F. Siblings, G. Other relatives, H. Friends?” The response alternatives were “Daily/Once a week/Once a month/Once a quarter/More rarely/Never/Not relevant.” Less severe social isolation was defined as living alone and not being in direct contact with relatives or friends more than once a week. Severe social isolation was defined as living alone and not being in direct contact with relatives or friends more than once a month.^29^

#### Health variables

Comorbidities were identified by several methods: self-reported to the study physician, through medical examination, and by reviewing medical records. Diseases included heart and lung disease, arthrosis, cancer, depression, and cognitive status. Heart diseases include myocardial infarction, angina pectoris, or arrhythmia. Lung diseases include chronic obstructive pulmonary disease, asthma, and tuberculosis. Arthrosis included joints in the back, hip, knees, thumb (CMC 1 joint), or big toe (MTP 1 joint). Cancer refers to all types of malignant tumors while depression covers both recent and past episodes. Cognitive status was assessed by the Mini-Mental State Examination (MMSE) measuring global cognitive function. The scale ranges from 0 to 30 points and cognitive impairment was set at ⩽24 points.^30^ We used the Swedish version of the original MMSE, which has been translated by the Swedish Association for Cognitive Disorders and is employed extensively in Sweden.^31^ In a previous study, the accuracy of the MMSE as a screening tool for dementia among older adults in a Swedish population using 23/24 points as the cutoff had a sensitivity of 87%, a specificity of 92%, and a positive predictive value of 69%.^32^

#### Sleep symptoms

Sleep disturbance was assessed by questions on sleep difficulties in the Comprehensive Assessment and Referral Evaluation Scale (CARE).^33,34^ CARE has previously been used to assess physical disability in older adults and, in addition to questions about sleep, includes questions about heart and lung disease and blood pressure. The sleep disturbance scale has shown construct validity^35^ as well as concurrent and predictive validity.^36^

Sleep disturbance included eight questions covering different sleep symptoms. Participants were asked if they suffered from difficulty falling asleep, took sleep medication, if their sleep was interrupted during the night, if they had difficulty staying asleep due to moods or tension, itching or pain, difficulty returning to sleep after waking at night, feeling tired and sleeping more than 2 h during the day, and waking early.^35^ The response alternatives were yes/no, and each positive response equaled 1 point; thus, the scale ranged from 0 to 8 points. Participants scoring ⩾4 points were considered to have a sleep disturbance, while those scoring <4 points were deemed to have a mild or no sleep disturbance (non-sleep disturbance group).^37,38^

#### Statistical analysis

Descriptive statistics of the study sample are presented in Table 1. Raw odds ratios and 95% confidence interval for each sleep symptom on the sleep disturbance scale and sleep disturbance (⩾4 symptoms) at baseline in relation to levels of loneliness and social isolation at re-examination are presented in Table 2.

**Table 1.** Description of the study sample based on sex, age group, marital status, education, financial status, smoking habits, heart disease, lung disease, arthrosis, cancer depression, cognitive impairment, loneliness, and social isolation at baseline, and sleep disturbance at baseline and re-examination, *N* = 2897.

Variables	Study sample *n* (%)	Internal missing *n* (%)
Sex		
Women	1612 (55.6)	0 (0)
Men	1285 (44.4)	
Age group (years)		
60–69	2045 (70.6)	0 (0)
70–79	372 (12.8)	
80+	480 (16.6)	
Age mean (SD)	67.4 (8.8)	
Marital status		
Married/cohabiting	1786 (62.1)	21 (0.7)
Unmarried/widowed/separated	1090 (37.9)	
Education		
Elementary school	1243 (44.3)	91 (3.1)
High school/college	1563 (55.7)	
Financial status		
Poor	140 (5.0)	91 (3.1)
Good	2666 (95.0)	
Smoking habits		
Non-smoker	1166 (40.2)	27 (0.9)
Former smoker	1191 (41.1)	
Current smoker	513 (17.7)	
Heart disease	562 (19.9)	78 (2.7)
Lung disease	324 (11.5)	76 (2.6)
Arthrosis	738 (26.2)	84 (2.9)
Cancer	384 (13.6)	78 (2.7)
Depression	493 (17.5)	81 (2.8)
Cognitive impairment		125 (4.3)
Mini-mental state examination		
0–24 p, cognitive impairment	265 (9.6)	
25–30 p, no cognitive impairment	2507 (90.4)	
Loneliness		
Never	1120 (40.3)	121 (4.2)
Single occasions	1310 (47.2)	
Recurring periods/constant	346 (12.5)	
Social isolation		
Not social isolated	2089 (72.1)	41 (1.4)
Less severe social isolation	641 (22.1)	
Severe social isolation	126 (4.3)	
Sleep disturbance (⩾4 sleep symptoms)		
Baseline examination	716 (25.6)	103 (3.6)
Re-examination	611 (23.7)	319 (11.0)

**Table 2.** Odds ratios of sleep symptoms and sleep disturbance at re-examination in relation to levels of loneliness and social isolation at baseline, *N* = 2897.

Loneliness/social isolation	Difficulty falling asleep	Using sleep medication	Sleep interrupted during the night	Difficulty staying asleep due to mood or tension	Difficulty staying asleep due to itching or pain	Difficulty returning to sleep after waking	Sleeping 2 h in the daytime	Waking early	Sleep disturbance ⩾4 sleep symptoms
Loneliness *n* (%)	664 (24.6)	354 (14.0)	2019 (80.2)	736 (29.5)	382 (15.3)	383 (15.3)	227 (9.1)	1424 (56.9)	583 (23.5)
	*n* = 2693	*n* = 2521	*n* = 2516	*n* = 2496	*n* = 2501	*n* = 2498	*n* = 2492	*n* = 2502	*n* = 2484
	OR (95% CI	OR (95% CI	OR (95% CI	OR (95% CI	OR (95% CI	OR (95% CI	OR (95% CI	OR (95% CI	OR (95% CI
Never	1	1	1	1	1	1	1	1	1
Occasionally	1.57	1.55	1.38	1.87	1.49	1.72	1.17	1.13	1.72
	(1.28–1.91)	(1.19–2.01)	(1.12–1.70)	(1.54–2.28)	(1.16–1.91)	(1.31–2.21)	(0.86–1.60)	(0.96–1.34)	(1.42–2.11)
Recurrent/constant	2.96	3.57	1.71	3.81	2.39	2.32	2.60	1.37	4.52
	(2.27–3.87)	(2.58–4.93)	(1.21–2.41)	(2.91–5.00)	(1.73–3.31)	(1.67–3.24)	(1.78–3.79)	(1.06–1.78)	(3.45–5.88)
Social isolation *n* (%)	679 (24.5)	367 (14.2)	2064 (80.0)	754 (29.1)	395 (15.4)	389 (15.2)	231 (9.0)	1460 (56.9)	598 (23.5)
	*n* = 2770	*n* = 2587	*n* = 2581	*n* = 2561	*n* = 2565	*n* = 2561	*n* = 2555	*n* = 2565	*n* = 2546
	OR (95% CI	OR (95% CI	OR (95% CI	OR (95% CI	OR (95% CI	OR (95% CI	OR (95% CI	OR (95% CI	OR (95% CI
No	1	1	1	1	1	1	1	1	1
Less severe	1.48	1.66	1.09	1.50	1.12	1.57	1.80	1.25	1.60
	(1.21–1.81)	(1.29–2.12)	(0.86–1.38)	(1.23–1.83)	(0.87–1.45)	(1.23–2.01)	(1.34–2.43)	(1.03–1.51)	(1.29–1.97)
Severe	1.58	2.21	1.14	1.78	1.47	1.78	1.84	1.12	1.96
	(1.06–2.35)	(1.40–3.51)	(0.64–1.88)	(1.19–2.65)	(0.91–2.39)	(1.10–2.87)	(1.02–3.31)	(0.76–1.67)	(1.29–2.97)

To identify the association with disturbed sleep at re-examination, a logistic regression model was constructed, and Nagelkerke *R*^2^ and Hosmer Lemeshow goodness of fit regression coefficients were calculated (Table 3). Independent variables from the baseline examination included in the multivariate logistic regression model were sex, age, marital status, education, financial status, smoking habits, heart disease, lung disease, arthrosis, cancer, depression, cognitive status, loneliness, social isolation, and sleep disturbance (at baseline). Only those participants who had complete data in all variables were included in the logistic regression model (*n* = 2411). To determine whether the population in the regression model differed from the study population (*n* = 2897), the two were compared regarding sex distribution and differences in mean age. The regression model was further tested for multicollinearity and none of the included variables showed a variance inflation factor >5.^39^

**Table 3.** A multivariate logistic regression model with independent variables from baseline examination and sleep disturbances at re-examination 6 years later as dependent variable, *n* = 2411.

Independent variables baseline	OR	95% CI	*p*-Value
Sex			
Men	1		
Women	1.55	1.22–1.99	<0.001
Age cohort (years)			
60	1		
70	1.28	0.91–1.80	0.112
80+	1.43	1.03–1.98	0.033
Marital status			
Married/cohabiting	1		
Unmarried/widowed/separated	0.74	0.50–1.10	0.139
Education			
Elementary school	1		
High school/university	0.93	0.73–1.17	0.531
Financial situation			
Poor	1		
Good	0.98	0.61–1.58	0.936
Smoking habits			
Non-smoker	1		
Former smoker	0.94	0.73–1.21	0.623
Current smoker	1.02	0.73–1.42	0.917
Heart disease (ref. no heart disease)	1.41	1.07–1.86	0.015
Lung disease (ref. no lung disease)	1.43	1.03–2.00	0.034
Arthrosis (ref. no arthrosis)	1.31	1.02–1.67	0.033
Cancer (ref. no cancer)	0.9	0.88–1.67	0.236
Depression (ref. no depression)	1.2	0.90–1.59	0.215
Mini-mental state examination			
0–24 p, cognitive impairment	1		
25–30 p, no cognitive impairment	−0.78	0.46–0.32	<0.001
Loneliness			
Never	1		
Occasionally	1.37	1.05–1.78	0.019
Recurring periods/constant	1.92	1.32–2.78	<0.001
Social isolation			
No	1		
Less severe	1.18	0.78–1.79	0.44
Severe	1.88	1.01–3.49	0.046
Sleep disturbances at baseline (⩾4 sleep symptoms)			
No	1		
Yes	9.83	7.77–12.42	<0.001

Nagelkerke *R*^2^ = 0.356, Hosmer Lemeshow goodness of fit, *χ*^2^ (df: 8, *n* = 2411) = 5.99, *p* = 0.647.

An attrition analysis was conducted to examine and compare the non-participants, that is, those who did not attend the re-examination (*n* = 1557), with the participants at the re-examination (*n* = 2897). The analysis was performed based on data collected at baseline in wave 1 (*n* = 2931) and wave 2 (*n* = 1523), a total of (*n* = 4454) individuals (Figure 1). The chi-squared test was used to determine any statistical differences between the groups, and the level of significance was set at *p* < 0.05 (Table 4). Data analysis was performed using SPSS for Windows, version 24.0 (IBM 211 Corporation, Armonk, NY, USA).

**Table 4.** Attrition analysis comparing non-participants with participants at baseline. The difference in distribution was analyzed with the chi-squared test.

Variable	Non-participants (*n* = 1557)		Participants (*n* = 2897)		*p*-Value
	*n*	%	*N*	%	
Sex					
Men	683/1557	43.9	1,285/2897	44.4	0.753
Women	874/1557	56.1	1612/2897	55.6	
Age mean, (SD)	76.5 (8.8)		67.4 (11.1)		<0.001
Age cohort (years)					
60	534/1557	34.3	2045/2897	70.6	<0.001
70	187/1557	12.0	372/2897	12.8	
80+	836/1557	53.7	480/2897	16.6	
Marital status					
Married/cohabiting	691/1458	47.4	1786/2876	62.1	<0.001
Unmarried/widowed/separated	767/1458	52.6	1090/2876	37.9	
Education					
Elementary school	779/1367	57.0	1243/2806	44.3	<0.001
High school/university	588/1367	43.0	1563/2806	55.7	
Financial situation					
Poor	84/1353	6.2	140/2806	5.0	0.103
Good	1269/1353	93.8	2666/2806	95.0	
Smoking habits					
Non-smoker	637/1434	44.4	1166/2870	40.6	<0.001
Former smoker	565/1434	39.4	1191/2870	41.5	
Current smoker	232/1434	16.2	513/2870	17.9	
Heart disease	526/1469	35.8	562/2819	19.9	<0.001
Lung disease	202/1467	13.8	324/2821	11.5	0.031
Arthrosis	421/1463	28.8	738/2813	26.2	0.076
Cancer	293/1471	19.9	384/2819	13.6	<0.001
Depression	303/1466	20.7	493/2816	17.5	0.012
Mini-mental state examination <25 p, cognitively impaired	377/1305	28.9	265/2772	9.6	<0.001
Loneliness					
Never	457/1262	36.2	1,120/2776	40.3	
Occasionally	562/1262	44.5	1,310/2776	47.2	<0.001
Recurring periods/constant	243/1262	19.3	346/2776	12.5	
Social isolation					
Not social isolated	874/1.400	62.4	2089/2856	73.1	
Less severe social isolation	377/1.400	26.9	641/2856	22.4	<0.001
Severe social isolation	149/1.400	10.6	126/2856	4.4	
Sleep disturbances at baseline 4 sleep symptoms					
No	314/1360	21.3	946/2804	33.7	<0.001
Yes	1046/1360	79.9	1860/2804	66.3	

#### Ethical considerations

The study was conducted in accordance with the Helsinki Declaration^40^ and approved by the regional ethics committee at Lund University from 2010 to 2012, registration no. LU 744-00. All participants provided written consent as well as permission to retrieve information from the National Patient Register and medical records. They were informed that they could withdraw from the study at any time.

## Results

A total of 2897 individuals were included in this study (Figure 1), of whom 1612 (55.6%, 95% CI: 53.8%–57.4%) were women and 1285 (44.4%, 95% CI: 42.6%–46.2%) men. The mean age at baseline was 67.4 years (SD = 8.8 years) and at re-examination 73.4 years (SD = 8.9 years). At baseline, recurring periods or constant feelings of loneliness were reported by 12.5% (95% CI: 11.3%–13.7%), severe social isolation by 4.3% (95% CI: 3.6%–5.1%), and less severe social isolation by 22.1% (95% CI: 20.6%–23.6%). Sleep disturbances were reported in 25.6% (95% CI: 24.0%–27.2%) at baseline and 23.7% (95% CI: 22.1%–25.3%) at re-examination (Table 1).

The symptom that showed the highest odds ratio at re-examination in relation to levels of loneliness at baseline was “difficulty staying asleep due to mood or tension,” recurrent/constant feelings of loneliness compared to never being lonely (OR: 3.81, 95% CI: 2.91–5.00). The symptom that showed the highest odds ratio at re-examination in relation to social isolation at baseline was “using sleep medication,” severe social isolation compared to not being socially isolated (OR: 2.21, 95% CI: 1.40–2.51) (Table 2). Furthermore, unadjusted odds ratios for sleep disturbance (⩾4 sleep symptoms) at re-examination in relation to loneliness at baseline was recurrent/constant feelings of loneliness compared to not being lonely (OR: 4.52, 95% CI: 4.45–5.88), and unadjusted odds ratio for sleep disturbance at re-examination in relation to social isolation at baseline was severe social isolation compared to not being socially isolated (OR: 1.96, 95% CI: 1.29–2.97) (Table 2).

Due to missing data in several variables, 2411 individuals were included in the multivariate logistic regression model, of whom 1319 (54.7%, 95% CI: 52.7%–56.7 %) were women and 1092 (45.3%, 95% CI: 44.7%–45.6%) were men. Their mean age was 66.9 years (SD = 8.4 years). The main variables predicting sleep disturbances at re-examination were sleep disturbance at baseline (OR: 9.83, 95% CI: 7.77–12.42), recurring periods/constant feelings of loneliness (OR: 1.92, 95% CI: 1.32–2.78), severe social isolation (OR: 1.88, 95% CI: 1.01–3.49), and female sex (OR: 1.55, 95% CI: 1.22–1.99) (Table 3).

The attrition analysis showed that non-participants were significantly older than the study sample and had a higher proportion of heart disease, cancer, depression, poorer cognitive function, recurrent/constant periods of loneliness, and severe social isolation (Table 4).

## Discussion

The aim of this follow-up study was to investigate whether loneliness and social isolation in a sample of older adults, mean age of 67.4 years at baseline examination, were associated with sleep disturbances at re-examination at a mean age of 76.4 years. We found that loneliness and severe social isolation were associated with increased odds of sleep disturbance at re-examination (mean time 6 years later) after adjusting for potential sociodemographic and health-related confounding factors.

Compared to our results, earlier research has partly pointed in the opposite direction. As previously mentioned, it was shown in a 6-year follow-up study that social isolation has an adverse effect on the quality of sleep, whereas loneliness does not.^23^ Others have reported that loneliness predicted poor sleep quality, while social isolation did not.^22^ When it comes to loneliness, a 7-year follow-up study found that loneliness predicted poor sleep,^41^ which also agrees with our results, and in a 3-year follow-up study, loneliness was shown to predict a decline in sleep adequacy.^42^ If one considers sleep disturbance as a measure that includes several symptoms, the present result partly agrees with one of our previous studies, in which we included 5804 participants with the intention of investigating the relationship between loneliness, social isolation, and symptom burden.^27^ In that study with the same level of loneliness but social isolation dichotomized into yes or no and not including sleep disturbance as a dependent variable, we found that loneliness, but not social isolation, was related to depressive, gastrointestinal-urinary, musculoskeletal, metabolic, cardiopulmonary, head, and tension symptoms.^27^

In terms of social isolation, less severe social isolation was reported by 22.2%, which is consistent with previous studies^29^ and did not significantly predict sleep disturbance. This might be explained by the fact that living alone and meeting friends or relatives once a week represents a level of social isolation that can be tolerated without negatively affecting sleep.

Regarding more pronounced or severe social isolation, previous studies have estimated the prevalence from 17% to 35%.^29,43^ In our study, no more than 4.3% were classified as suffering from severe social isolation. It is possible that the definition used in our study is too narrow and that one can experience severe social isolation despite having contact with relatives and friends at least once a month. In the operationalization of being isolated, we did not consider involvement in different social groups or organizations, as participation in such contexts may be impossible due to impaired health or insufficient communication ability.

We did not ask the participants about indirect social contacts, for example, via digital media. Previous studies have shown that social networking sites and smartphones can dramatically increase the opportunities for social contacts,^44^ thereby possibly reducing the negative impact of physical social isolation. Nevertheless, some older adults may feel excluded, as modern technology can be perceived as difficult to master, while others may find it easier to use digital media to stay in contact with people.^45^ An explanation could also be that similar to loneliness, issues of severe social isolation may be associated with negative emotions, and for that reason, some underreporting cannot be ruled out.

Bed-sharing is another topic that should be mentioned in connection with social isolation and sleep disturbance. Objective measures made by polysomnography actigraphy have shown an association between bed-sharing and multiple wakening, interpreted as impaired sleep quality. By contrast, subjective measurements made using questionnaires describe bed-sharing as something positive for the quality of sleep, which is explained by the psychological benefits that bed-sharing can have; for example, if something serious should happen you are not alone.^46^ However, without investigating the possible effects of bed-sharing in terms of sleep disturbance, the present study showed that when subjectively measured, severe socially isolated participants have worse sleep. A hypothetical question is whether the result would have been different if we had the opportunity to objectively measure sleep symptoms.

Loneliness was assessed by how often the participants had feelings of loneliness, not by how strongly they experienced such feelings. We cannot rule out the possibility that temporary but intense feelings of loneliness can have the same negative effects as prolonged periods of loneliness. Although we did not consider loneliness in a qualitative sense, we found a significant association between all levels of loneliness and sleep disturbances.

The estimate of the percentage that claims to be lonely varies a great deal between different surveys; from 19% to 32% in studies from North America and from 12% to 56% in studies from Europe.^47^ As for social isolation, some of the explanations for loneliness may be related to methodology, such as the use of different scales, single questions or multi-question instruments, how the data were collected, the size of the study sample,^48^ or geographical, cultural, or gender differences in the causes of loneliness, that is, physical and mental health, sociodemographics, or how loneliness is perceived.^3,47,48^

Insomnia is common in adults, although the reported prevalence differs greatly between studies. Based on the definition of insomnia and the methodological approach, the prevalence varies from 5% to 50%.^49^

About 50% of individuals over the age of 60 years have been reported to suffer from a sleep disorder,^6,9^ which is partly explained by the fact that aging is associated with a higher prevalence of physical and mental illness, polypharmacy, and social factors that can affect sleep.^9,50^ However, the prevalence of sleep disturbances in the present study reported by about 25% of the participants at both baseline and re-examination is consistent with previous studies with similar populations using ⩾4 symptoms indicating sleep disturbance.^37,38^ Similar results have also been shown in another Swedish study, where about 25% of the men and 33% of the women aged 60–84 years suffered from insomnia symptoms, that is, difficulties falling asleep and/or staying asleep.^51^

Although the average age at re-examination was 6 years older and the percentage of 70- and 80-year-olds had increased, the proportion reporting sleep disturbances was approximately the same. This might be because the causes of sleep disturbances may vary in different age groups. Social factors, such as impending retirement with changes in social networks and financial situation, are more pronounced in the 60-year-old group,^52^ while illness and disability are more prevalent in older age groups.^53^ A question is whether reducing feelings of loneliness would improve the quality of sleep, or health in general, and if so, how it could be done.^53^

Previous interventions have employed different strategies, such as improving social skills, strengthening the social network, creating opportunities for social interactions,^54^ and counteracting maladaptive social cognition, that is, inappropriate expectations, thoughts, and feelings about relationships.^55^ Such approaches have been able to reduce loneliness and social isolation but, to the best of our knowledge, evidence of effective interventions that improve sleep habits by reducing feelings of loneliness has not been reported. Thus, further studies are warranted on reducing loneliness and social isolation, as well as the importance of better sleep habits.

Although this study primarily examined loneliness and social isolation as potential predictors of sleep disturbances, the reverse relationship is certainly plausible. A previous study of American participants aged 65 and older demonstrated how loneliness and sleep disturbances interacted over time, that is, poorer sleep led to loneliness, which, in turn, resulted in poorer sleep in a vicious circle.^56^

### Strengths

A strength of the present study was that the participants were randomly selected from the population register, covering both rural and urban areas. Only standardized questionnaires were used, and all examinations were carried out by trained personnel. To reduce selection bias, home visits were made to those unable to visit the study centers. Help was offered to participants who had difficulties answering the questionnaires due to language problems, visual impairment, or other disabilities.

### Limitations

The study design can be seen as a limitation in that data on sleep disturbance, social isolation, and loneliness were only collected on two occasions. A study with more collection occasions, where fluctuations in loneliness might have shown corresponding changes in sleep disturbance, would have produced a more nuanced result.

A further limitation might be the lack of a power analysis. The number of individuals invited to take part in the studies was calculated based on the clinic’s capacity and the time at our disposal.

Although loneliness and social isolation represent different constructs, they can occur simultaneously. In such cases, and even if the correlation between the constructs is found to be low, an additive or synergistic effect cannot be ruled out.^57^ As the aim of this study was limited to investigating whether levels of loneliness and/or social isolation were associated with sleep disturbances, we did not evaluate any possible interaction between them.

For the same reason, we have not investigated how loneliness or social isolation may affect sleep quality but it should be mentioned that previous studies have shown that feelings of loneliness are in themselves stressful experiences and that perceived stress may be a link between feelings of loneliness and poorer sleep quality.^22^

We used a single-item question to assess loneliness. A limitation of such an assessment is that it assumes the participants understand the concept of loneliness. To address this, the following information was provided in the questionnaire: “By loneliness, we mean the specific feeling of being lonely, and not whether you are with other people or not.” In addition, it can be advantageous to use a single-item question, as when the meaning is straightforward and thoroughly explained, it can be easily interpreted by the participants.^58^ Nevertheless, using a single direct question can also be counterproductive. Identifying oneself as lonely can evoke feelings of discomfort or even stigma, which can lead to underreporting.^48^

Another possible limitation was that two of the most common causes of sleep disturbances in older adults, apnea and restless legs, were not included in the sleep disturbance scale. However, the intention was not to study possible relationships between loneliness/social isolation and specific diagnoses but to identify sleep disturbances as a measure based on described sleep symptoms.

There is an ongoing discussion about whether the included risk factors are correctly chosen. We have tried as far as possible, based on previous studies, to select relevant risk factors that can contribute to sleep symptoms, that is, marital and economic status, education level^49^ and from a geriatric perspective, health conditions that are common in older adults. However, a limitation is that environmental factors associated with sleep problems, such as disturbing noise or room temperature, were not considered.^59^

Although home visits were made to avoid selection bias, the attrition rate amounted to 35%. As might be expected in a follow-up study among older adults, non-participants were both older and in poorer health compared to the study sample. In addition, the proportion reporting recurrent/constant periods of loneliness and social isolation was significantly greater in the non-participant group, a selection bias that may have resulted in reduced associations between recurrent periods of/constant loneliness or social isolation and sleep disturbances.

An additional limitation may be that only participants with complete data in the various covariates were included in the multivariate logistic regression model (Table 3). In a comparison between participants included in the regression model (*n* = 2411) and the entire study population (*N* = 2897) regarding sex distribution and mean age, the differences as presented in paragraphs 1 and 3 of the results section were found to be small and bias can therefore probably be ruled out.

## Conclusion

In this Swedish cohort study, recurrent or constant feelings of loneliness as well as severe social isolation were associated with sleep disturbances among individuals aged 60 years and over. Thus, it could be important for physicians and other medical personnel to be aware of the potential influence of loneliness and social isolation when assessing sleep disturbances in older adults. Interventions to reduce either recurrent periods of or constant loneliness may play a role in the care of patients reporting sleep disturbances.

## Supplemental Material

sj-docx-1-smo-10.1177_20503121231222823 – Supplemental material for The association between loneliness, social isolation, and sleep disturbances in older adults: A follow-up study from the Swedish good aging in Skåne project Click here for additional data file. Supplemental material, sj-docx-1-smo-10.1177_20503121231222823 for The association between loneliness, social isolation, and sleep disturbances in older adults: A follow-up study from the Swedish good aging in Skåne project by Henrik Ekström, Markus Svensson, Sölve Elmståhl and Lena Sandin Wranker in SAGE Open Medicine
