# Supplementary material for: The association between loneliness, social isolation, and sleep disturbances in older adults: A follow-up study from the Swedish good aging in Skåne project
Source: SAGE Open Med. 2024 Jan 18;12:20503121231222823. doi: 10.1177/20503121231222823 (PMC10798090; doi:10.1177/20503121231222823)
Supplement: sj-docx-1-smo-10.1177_20503121231222823 – Supplemental material for The association between loneliness, social isolation, and sleep disturbances in older adults: A follow-up study from the Swedish good aging in Skåne project [file sj-docx-1-smo-10.1177_20503121231222823.docx]

**Supplementary material**

**Sleep disturbance scale**

| **Questions about your sleep** |
| --- |
| **162. Do you have difficulty falling asleep? 1 Yes 2 No** |
| **163. Do you take or are you dependent on medicine to be able to sleep? 1 Yes 2 No** |
| **164. Do you wake up during the night? 1 Yes 2 No** |
| **165. Do you have difficulty falling asleep/staying asleep due to mood or tension? 1 Yes 2 No** |
| **166. Do you have difficulty sleeping because of pain or itching? 1 Yes 2 No** |
| **167. Are you unable to go back to sleep after waking up at night? 1 Yes 2 No** |
| **168. Do you wake up early? 1 Yes 2 No** |
| **169. Do you feel tired and sleep more than two hours during the day? 1 Yes 2 No** |
